# Inherent Plasticity of Brown Adipogenesis in White Fat of Mice Allows for Recovery from Effects of Post-Natal Malnutrition

**DOI:** 10.1371/journal.pone.0030392

**Published:** 2012-02-24

**Authors:** Leslie P. Kozak, Robert A. Koza, Rea Anunciado-Koza, Tamra Mendoza, Susan Newman

**Affiliations:** 1 Institute of Animal Reproduction and Food Research of the Polish Academy of Sciences, Olsztyn, Poland; 2 Pennington Biomedical Research Center, Baton Rouge, Louisiana, United States of America; University of Cincinnati, United States of America

## Abstract

Interscapular brown adipose tissue (iBAT) is formed during fetal development and stable for the life span of the mouse. In addition, brown adipocytes also appear in white fat depots (wBAT) between 10 and 21 days of age in mice maintained at a room temperature of 23°C. However, this expression is transient. By 60 days of age the brown adipocytes have disappeared, but they can re-emerge if the adult mouse is exposed to the cold (5°C) or treated with β3-adrenergic agonists. Since the number of brown adipocytes that can be induced in white fat influences the capacity of the mouse to resist the obese state, we determined the effects of the nutritional conditions on post-natal development (birth to 21 days) of wBAT and its long-term effects on diet-induced obesity (DIO). Under-nutrition caused essentially complete suppression of wBAT in inguinal fat at 21 days of age, as indicated by expression of *Ucp1* and genes of mitochondrial structure and function based upon microarray and qRT-PCR analysis, whereas over-nutrition had no discernible effects on wBAT induction. Surprisingly, the suppression of wBAT at 21 days of age did not affect DIO in adult mice maintained at 23°C, nor did it affect the reduction in obesity or cold tolerance when DIO mice were exposed to the cold at 5°C for one week. Gene expression analysis indicated that mice raised under conditions that suppressed wBAT at 21 days of age were able to normally induce wBAT as adults. Therefore, neither severe hypoleptinemia nor hypoinsulinemia during suckling permanently impaired brown adipogenesis in white fat. In addition, energy balance studies of DIO mice exposed to cold indicates that mice with reduced adipose stores preferentially increased food intake, whereas those with larger adipose tissue depots preferred to utilize energy from their adipose stores.

## Introduction

Interscapular brown adipose tissue (iBAT) in mice first appears during the last days of gestation, matures during the sucking period and remains at a relatively stable level throughout the life of the animal [1], [2], [3]. In contrast, brown adipocytes, which are induced in white fat depots by cold exposure or treatment with β3-adrenergic agonists in adults (wBAT), first appear transiently in the both subcutaneous and visceral fat depots at approximately 21 days of age [3]. Since the mice are maintained at normal room temperature (23°C) at 21 days of age, this induction seems to be independent of enhanced adrenergic signaling that normally mediates cold-induced thermogenesis in adult mice [4], [5]. Whereas the differentiation of iBAT occurs during fetal development, consistent with a function in maintenance of body temperature in the newborn, the physiological function of wBAT in white fat depots at weaning suggests that wBAT has a role in body weight regulation; but, this is mere speculation. Increases in wBAT, induced in several transgenic mice, generally cause resistance to diet-induced obesity [6], [7]. While providing proof of principle that increases in wBAT can reduce obesity; nevertheless, these models do not clarify the normal physiological function of wBAT. In a genetic model based upon allelic variation in the induction of wBAT in recombinant inbred strains (RI; AXB, BXA) of mice upon adrenergic stimulation, the diet-induced obesity in mice fed a high fat diet at a room temperature of 23°C does not correlate with the potential induction of wBAT. However, when RI mice with induced DIO are stimulated adrenergically by β3-adrenergic agonists, RI mice with a greater capacity for induction of wBAT lose more adipose tissue [8].

In an experiment aimed at determining the developmental basis for non-genetic variation in diet-induced obesity in C57BL/6J mice, we adapted published protocols to raise mice from birth to weaning under-conditions of over- and under-nutrition [9], [10], [11]. We showed that restricted feeding (50% of the food consumed by nursing dams fed ad libitum) of the nursing dam from birth to 21 days of age (LUN group) led to a severe restriction of adipose tissue mass development that strongly affects the capacity for diet-induced obesity in adult mice [11]. Of particular interest was that the under-nutritional protocol (LUN) reduced the expression of a set of genes, associated with the cytoskeleton and caveolae structures, in the inguinal fat of 10 day old mice in proportion to their capacity for adipose tissue expansion.

Since the adipocytes or resident progenitors of the inguinal fat in a the suckling mouse will also give rise to the brown adipocytes that appear at 21 days of age [3], we used K-means cluster analysis of the microarray data to determine whether the differentiation of the wBAT would be affected by under-nutrition during early post-natal development. Having observed that under-nutrition causes a profound suppression of wBAT differentiation at 21 days of age, we assessed the consequences of suppression of the brown adipocyte differentiation program at 21 days on the capacity for diet-induced obesity in mice fed a high fat diet from 8 to 16 weeks of age. If the brown adipocytes in the white fat depots are involved in the regulation of body weight, then suppression of brown adipocyte differentiation by under-nutrition during early post-natal development, that is, at 21 days of age, may reduce the capacity for diet-induced thermogenesis and result in increased diet-induced obesity in those adult mice that were under-nourished during the lactation period. The results suggest that DIO was not enhanced in adult mice with a history of under-nutrition compared to control mice and that the induction of brown adipocytes in white fat depots of adult mice by exposure to an ambient temperature of 4°C for one week was not impaired by under-nutrition during early post-natal development.

## Results

### Suppression of gene expression during wBAT differentiation by under-nutrition during early development

To identify the regulatory pathways associated with environmentally-dependent and genetically-independent modulation of adipose tissue expansion during early development, microarray analyses were performed on RNA from inguinal fat of C57BL/6J mice in the Control, LUN and LON groups at 5,10, 21, 56 and 112 days of age (for nutritional protocol see [11] ). K-means cluster and Venn analysis revealed a set of genes that were expressed in parallel with the rate of fat mass accumulation, that is, expression reach their highest levels at 5 and 10 days of age and then again at 112 days of age following 8 weeks on a high fat diet, whereas the lowest levels of expression were at 21 and 56 days of age [11]. The major genes present in this set encoded components of the caveolae and cytoskeleton, which have previously been implicated in fat mass expansion [12], [13], [14].

**Figure 1:**
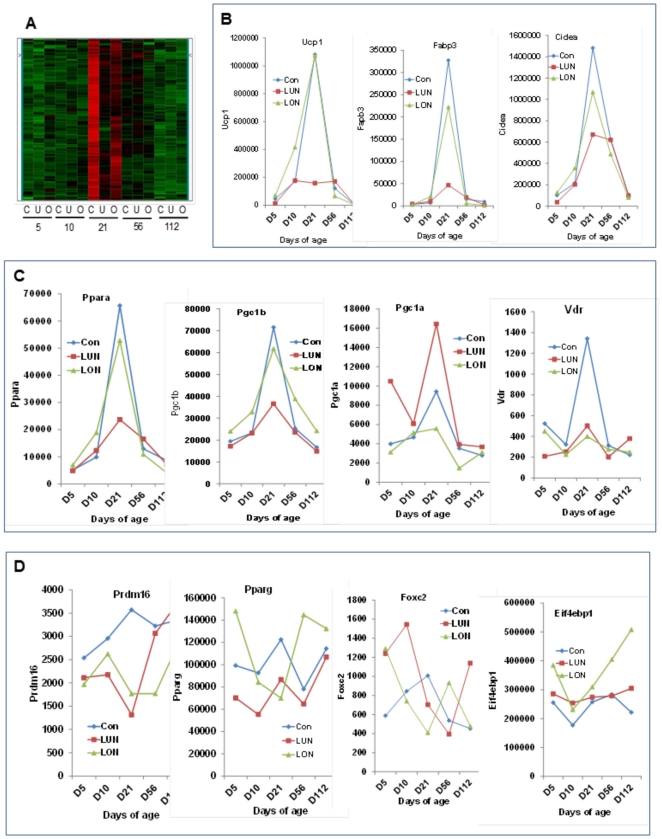
Genomic analysis of regulatory factors for wBAT induction during early development. **A**. Heat map of changes in gene expression in a K-means cluster of 180 genes with an *Ucp1*-like profile in inguinal fat of mice at 5, 10, 21, 56 and 112 days of age under control (C), under-nutrition (U) and over-nutrition (O) conditions from birth to 21 days of age. From 21 days of age until 56 days of age all mice were fed a low fat chow diet and from 56 to 112 days of age they were fed a high fat diet as described [11]. **B**. Expression profiles of *Ucp1*/BAT marker genes; **C**. a set of genes encoding regulatory factors with profiles similar to *Ucp1*; **D**. A set of putative regulatory factors for *Ucp1* expression in BAT in which their profiles do not correspond to that of *Ucp1*.

**Figure 2:**
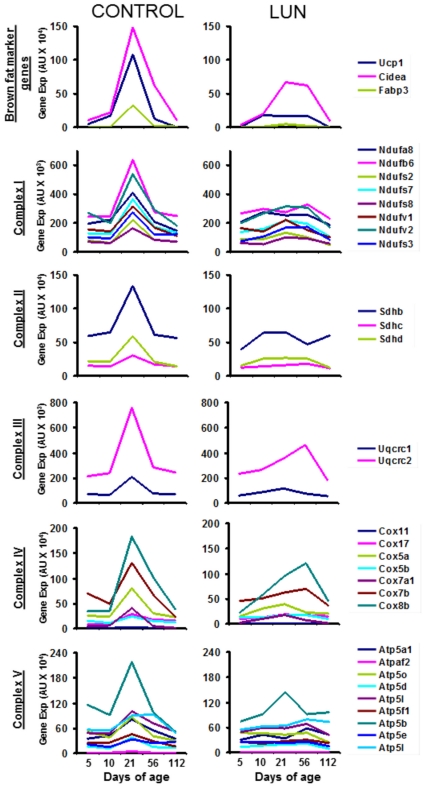
Genomic analysis of genes of respiration and oxidative phosphorylation. Comparison of expression profiles of genes in the Ucp1 K-means cluster that encode components of the respiration, Complexes I to IV, and ATP synthase, Complex V, during control (graphs on the left) and under-nutrition (graphs on the right) dietary conditions as described in the Legend to Figure 1 and EXPERIMENTAL PROCEDURES.

During the course of the analysis of microarray expression data for adipose tissue expansion we uncovered a K-means cluster of genes with patterns of expression similar to *Ucp1* (Fig. 1A). These genes could be associated with the developmental induction of brown adipocytes in white fat depots. Under standard control diet these genes showed a peak of expression at 21 days of age, consistent with previous studies based upon qRNA, immunoblot analysis of protein markers and immunohistology of brown fat adipogenesis [3]. A striking feature of the expression pattern in the *Ucp1*- gene cluster was the huge reduction in gene expression in mice from the under-nourished group (LUN). BAT marker genes especially suppressed by under-nutrition were *Ucp1*(7 fold), *Fabp3*(7-fold) and *Cidea* (2-fold) (Fig. 1B). Although not so severely suppressed as *Ucp1*, the major class of genes within this *Ucp1*-gene cluster encoded components of mitochondrial structure and metabolism, including those of electron transport and oxidative phosphorylation, the TCA cycle, fatty acid oxidation and reactive oxygen species (180 genes were in the K-means cluster of which ∼30% encode proteins located in the mitochondria). A partial list of the mitochondrial associated genes in the cluster is shown in Table 1. One interpretation of the gene expression data is that the transient induction of the brown fat differentiation program at 21 days of age is accompanied by a doubling of the mitochondria content of inguinal fat, evident by the 2 fold increase in gene expression, which normally occurs under control nutritional conditions. The suppression of induction in mitochondrial gene expression in the under-nutrition group essential results in a flat profile for the virtually all of the genes associated with electron transport and oxidative phosphorylation (Figure 2).

**Table 1:** Effects of malnutrition on genes expressed in mitochondria of inguinal fat.

	Gene_Symbol	Gene_Name	D21-CONT	D21-LUN	D21-LON	C 21/LUN21	C21/LON21
Brown fat specific genes							
	Ucp1	uncoupling protein 1 (mitochondrial, proton carrier)	1.08E+06	1.58E+05	1.07E+06	6.84	1.01
	Cidea	cell death-inducing DNA fragmentation factor	1.48E+06	6.70E+05	1.07E+06	2.21	1.39
Mitochondrial ETC							
Complex I	Ndufa8	NADH dehydrogenase (ubiquinone) 1 alpha subcomplex, 8	4.09E+05	2.56E+05	3.13E+05	1.60	1.31
	Ndufb6	NADH dehydrogenase (ubiquinone) 1 beta subcomplex, 6	6.38E+05	2.75E+05	4.43E+05	2.32	1.44
	Ndufs2	NADH dehydrogenase (ubiquinone) Fe-S protein 2	2.23E+05	1.35E+05	1.49E+05	1.65	1.50
	Ndufs7	NADH dehydrogenase (ubiquinone) Fe-S protein 7	3.66E+05	2.17E+05	2.11E+05	1.68	1.73
	Ndufs8	NADH dehydrogenase (ubiquinone) Fe-S protein 8	1.67E+05	1.03E+05	1.08E+05	1.63	1.55
	Ndufv1	NADH dehydrogenase (ubiquinone) flavoprotein 1	3.18E+05	2.22E+05	3.05E+05	1.43	1.04
	Ndufv2	NADH dehydrogenase (ubiquinone) flavoprotein 2	5.42E+05	3.18E+05	3.04E+05	1.70	1.78
	Ndufs3	NADH dehydrogenase (ubiquinone) Fe-S protein 3	2.78E+05	1.70E+05	1.49E+05	1.64	1.86
Complex II	Sdhb	succinate dehydrogenase complex, subunit B	1.33E+06	6.47E+05	8.81E+05	2.06	1.51
	Sdhc	succinate dehydrogenase complex, subunit C	3.09E+05	1.63E+05	2.42E+05	1.90	1.28
	Sdhd	succinate dehydrogenase complex, subunit D	5.94E+05	2.71E+05	3.65E+05	2.19	1.63
Complex III	Uqcrc1	ubiquinol-cytochrome c reductase core protein 1	2.06E+05	1.19E+05	1.37E+05	1.74	1.51
	Uqcrc2	ubiquinol cytochrome c reductase core protein 2	7.59E+05	3.58E+05	5.79E+05	2.12	1.31
Complex IV	Cox11	COX11 homolog, cytochrome c oxidase assembly protein	2.22E+04	1.40E+04	1.74E+04	1.58	1.28
	Cox17	cytochrome c oxidase, subunit XVII assembly protein	2.90E+05	1.88E+05	2.55E+05	1.54	1.14
	Cox5a	cytochrome c oxidase, subunit Va	7.98E+05	3.95E+05	4.75E+05	2.02	1.68
	Cox5b	cytochrome c oxidase, subunit Vb	2.44E+05	1.81E+05	1.85E+05	1.35	1.32
	Cox7a1	cytochrome c oxidase, subunit VIIa 1	4.12E+05	1.79E+05	4.59E+05	2.30	0.90
	Cox7b	cytochrome c oxidase subunit VIIb	1.31E+06	6.29E+05	8.77E+05	2.09	1.50
	Cox8b	cytochrome c oxidase, subunit VIIIb	1.83E+06	9.50E+05	1.35E+06	1.92	1.36
Complex V	Atp5d	ATP synthase, H+ transporting, mitochondrial F1 complex	3.86E+05	2.01E+05	3.01E+05	1.92	1.28
	Atp5e	ATP synthase, H+ transporting, mitochondrial F1 complex	3.32E+05	2.35E+05	3.71E+05	1.42	0.89
	Atpaf2	ATP synthase mitochondrial F1 complex assembly factor 2	3.97E+04	1.92E+04	3.20E+04	2.07	1.24
Miscellaneous ET proteins							
	Etfb	electron transferring flavoprotein, beta polypeptide	4.15E+05	2.57E+05	2.50E+05	1.61	1.66
Fatty acid metabolism							
	Acss1	acyl-CoA synthetase short-chain family member 1	1.81E+04	1.24E+04	1.61E+04	1.46	1.13
	Dci	dodecenoyl-Coenzyme A delta isomerase	4.51E+05	2.80E+05	2.71E+05	1.61	1.66
	Fabp3	fatty acid binding protein 3, muscle and heart	3.27E+05	4.64E+04	2.22E+05	7.05	1.47
	Gyk	glycerol kinase	1.57E+04	7.19E+03	2.02E+04	2.19	0.78
	Hadhb	hydroxyacyl-Coenzyme A dehydrogenase	2.03E+05	1.01E+05	1.88E+05	2.01	1.08
	Hadhsc	L-3-hydroxyacyl-Coenzyme A dehydrogenase, short chain	3.56E+05	2.18E+05	2.45E+05	1.63	1.45
	Acaa2	acetyl-Coenzyme A acyltransferase 2	3.30E+05	1.61E+05	3.21E+05	2.05	1.03
	Acad8	acyl-Coenzyme A dehydrogenase family, member 8	2.34E+04	1.65E+04	1.80E+04	1.41	1.30
	Acads	acyl-Coenzyme A dehydrogenase, short chain	9.82E+04	5.80E+04	8.30E+04	1.69	1.18
	Acadvl	acyl-Coenzyme A dehydrogenase, very long chain	1.42E+06	7.50E+05	1.03E+06	1.90	1.38
	Cpt1b	carnitine palmitoyltransferase 1b, muscle	1.66E+04	7.74E+03	1.29E+04	2.14	1.28
	Mlycd	malonyl-CoA decarboxylase	4.25E+04	2.97E+04	3.30E+04	1.43	1.29
	Slc25a20	mitochondrial carnitine/acylcarnitine translocase	7.81E+04	5.07E+04	9.68E+04	1.54	0.81
TCA cycle							
	Aco1	aconitase 1	2.16E+04	1.49E+04	1.82E+04	1.44	1.18
	Aco2	aconitase 2, mitochondrial	8.56E+05	4.64E+05	5.52E+05	1.84	1.55
	Idh3g	isocitrate dehydrogenase 3 (NAD+), gamma	3.61E+05	2.40E+05	2.80E+05	1.51	1.29
	Suclg1	succinate-CoA ligase, GDP-forming, alpha subunit	6.35E+04	4.00E+04	7.33E+04	1.59	0.87
	Suclg2	succinate-Coenzyme A ligase, GDP-forming, beta subunit	5.85E+04	4.19E+04	4.82E+04	1.39	1.21
	Mdh2	malate dehydrogenase 2, NAD (mitochondrial)	1.03E+06	6.74E+05	9.00E+05	1.53	1.14
	Ogdh	oxoglutarate dehydrogenase (lipoamide)	3.04E+05	1.19E+05	3.26E+05	2.56	0.93
	Pcca	propionyl-Coenzyme A carboxylase, alpha polypeptide	3.29E+05	1.66E+05	2.15E+05	1.98	1.53
	Mcee	methylmalonyl CoA epimerase	1.92E+05	1.01E+05	1.25E+05	1.91	1.53
Mitochondrial protein transporters							
	Timm17a	translocase of inner mitochondrial membrane 17a	9.24E+04	5.39E+04	7.52E+04	1.71	1.23
	Timm44	translocase of inner mitochondrial membrane 44	2.29E+04	1.87E+04	1.70E+04	1.22	1.34
	Tomm40l	translocase of outer mitochondrial membrane 40 homolog-like	3.67E+04	2.62E+04	3.08E+04	1.40	1.19
Reactive oxygen species							
	Prdx3	peroxiredoxin 3	4.18E+05	2.85E+05	4.19E+05	1.46	1.00
	Sod2	superoxide dismutase 2, mitochondrial	3.62E+05	1.57E+05	2.48E+05	2.31	1.46
	Txn2	thioredoxin 2	7.79E+04	4.73E+04	5.54E+04	1.65	1.40
Mitochondrial protein synthesis							
	Mrpl16	mitochondrial ribosomal protein L16	5.67E+04	3.20E+04	4.20E+04	1.78	1.35
	Mrpl43	mitochondrial ribosomal protein L43	8.63E+04	4.73E+04	6.99E+04	1.82	1.23
	Mrpl9	mitochondrial ribosomal protein L9	6.15E+04	3.60E+04	4.57E+04	1.71	1.34
	Mrps36	mitochondrial ribosomal protein S36	3.25E+05	1.60E+05	2.96E+05	2.03	1.10

Values in the columns D21-CON, D21-LUN and D2-1LON are teg average for data from 3 microarrys for each nutrtional group at 21 days of age. In the 2 columns on the right, C21 is control diet at day 21; LUN21 is under-nutrition at day 21 and LON21 is over-nurtion at day 21.

Evident also in Figure 2 is the remarkable coordinated suppression in the isoforms of the complexes of mitochondrial respiratory chain, suggesting that the overall brown adipocyte differentiation program, which includes brown fat specific genes i.e *Ucp1*, as well as the large repertoire of genes of the mitochondria, fails to occur during lactation under-nutrition. The gene expression data in Figure 2 is based upon the K-means cluster illustrated in Figure 1A. This cluster analysis does not contain all of the genes encoding components of the respiratory complexes for analytical reasons. That is, inspection of the complete microarray gene expression lists indicates that the other isoforms associated with the respiratory complexes were similarly suppressed by under-nutrition, but the Z-scores for these genes failed to include them in the clusters. From the perspective of the relation of *Ucp1* expression to limit the development of excess adiposity in state of positive energy balance, it is noteworthy that over-nutrition by feeding dams a high fat diet and reducing the number of pups to 4 per dam did not significantly increase expression of *Ucp1* or any of the genes of mitochondrial function compared to the control diet (Table 1). Accordingly, a highly positive energy balance during post-natal development does not enhance expression of genes of brown fat structure and function. Finally, under-nutrition (LUN) did not significantly affect the classical markers of white adipogenesis, that is, expression of lipoprotein lipase, the cytoplasmic NAD-linked glycerol-3-phosphate dehydrogenase, stearoyl-CoA desaturase 1, FABP4, adiponectin, adiponectin receptors 1 and 2 and resistin are not significantly suppressed at post-natal day 21 or for that matter at other days of age (see data in GEO repository, accession number GSE19809).

### Under-nutrition suppresses selective putative regulatory pathways associated with *Ucp1* expression and wBAT differentiation

In this study the wBAT differentiation program has been submitted to a microarray analysis of gene expression involving the interaction of developmental and nutritional variables that generated 15 independent experimental groups. Each group was composed of 12 mice from which RNA was isolated, pools were constructed with the equal aliquots of RNA from each mouse and then microarrays were performed on each RNA pool in triplicate. Regression analysis of the quantification of RNA expression for 8 genes by qRT-PCR vs microarray data obtained from the ABI 1700 system showed that with one exception the correlation coefficient R ranges between 0.82 and 1.00 with R for the combined analyses equal to 0.92 [11]. Thus, a high degree of confidence can be given to the quantitative microarray data. In the preceding section we have shown how diet and development have interacted to create highly coordinate expression patterns for components of functional mitochondria and gene products unique to the brown adipocyte, that is, *Ucp1* and respiratory complexes. What can the variation in gene expression in this nutrition study reveal about the regulatory factors of wBAT development?

The major research strategy towards the identification of regulatory components, both transcriptional and signaling, for induction of the brown fat differentiation program has been based upon correlations of transgenic, over-expression of a putative factor, for example FOXC2 and PRDM16 by the aP2 promoter, or from the appearance of the brown fat phenotype in KO mice. Approximately 20 genes have been shown to affect the wBAT phenotype using these strategies [15]. Accordingly, a comparison of changes in transcription factors implicated from gene targeting and over-expression in transgenic mice as well as those from genetic QTL studies will provide an independent test of the significance of nutrition environment on the differentiation of wBAT. As shown in Fig. 1B, the effects of under-nutrition on the expression of marker genes of wBAT differentiation are striking. The expression profiles of 8 regulatory factors previously linked to wBAT induction are shown in Fig. 1C and Fig. 1D. Only *Ppara* and *Ppargc1b* showed strong correlations with *Ucp1* expression as evident from the changes in expression from both a developmental and nutrition perspective. The strong similarities in profiles for these factors to *Ucp1* and the correlation analysis of expression data (correlation coefficients of R = 0.967 and 0.925 for *Ppara* and *Ppargc1b*, respectively) suggest the function of these 2 factors are strongly linked to induction of wBAT at 21 days of age (Table 2). *Pparg* and *Ppargc1a*, have expression profiles and the high levels of expression indicating that they have other functions linked to white fat development and the nutritional environment. Other factors, such as *Prdm16*, *Foxc2* and *Rbl1* have RNA profiles and expression levels that do not appear to relate to the profile of either brown or white fat development. However, this study does not address whether the genes in Fig. 1D may play critical roles in the early stages of wBAT differentiation at the progenitor or precursor stages.

**Table 2:** Correlation analysis between Ucp1 expression and that of its putative regulatory factors.

	Prdm16	Foxc2	RbI1	Rb1	Pparg	Ppara	Ppargc1a	Ppargc1b	Eif4ebp1	Vdr	Prkar2b	Dio2	*Eif4ebp2*	Ucp1
Prdm16	1.00													
Foxc2	−0.16	1.00												
RbI1	−0.13	0.39	1.00											
Rb1	−0.23	0.34	0.48	1.00										
Pparg	0.085	0.062	−0.096	0.49	1.00									
**Ppara**	0.011	−0.13	−0.089	−0.27	−0.096	1.00								
Ppargc1a	−0.38	0.16	−0.37	−0.35	−0.34	0.37	1.00							
**Ppargc1b**	−0.15	−0.12	−0.069	−0.033	0.082	**0.95**	0.30	1.00						
Eif4ebp1	−0.22	−0.025	0.18	0.60	0.56	−0.18	−0.27	0.019	1.00					
**Vdr**	0.21	0.13	−0.14	−0.20	0.29	0.74	0.36	0.70	−0.12	1.00				
Prkar2b	−0.064	−0.31	0.051	0.49	0.55	0.26	−0.32	0.44	0.73	0.077	1.00			
Dio2	0.26	0.12	0.15	0.34	0.21	−0.16	−0.32	−0.12	0.65	−0.15	0.46	1.00		
Eif4ebp2	−0.15	−0.022	0.62	0.59	0.26	0.11	−0.51	0.25	0.59	−0.074	0.62	0.54	*1.00*	
**Ucp1**	−0.006	−0.13	0.061	−0.18	−0.15	**0.97**	0.24	**0.93**	−0.19	**0.64**	0.25	−0.12	*0.27*	1.00

Table 1 also has gene expression data on the mice that were in the over-nutrition protocol (LON). One would predict that if a high fat obesogenic environment, such as that in the LON protocol, induced brown fat thermogenesis, then the *Ucp1*-K-means cluster would have many genes with higher expression in the LON mice than in mice under control dietary conditions. Inspection of the ratio C21/LON21 of Table 1 does not indicate that expression of BAT associated genes was higher in the LON21 group. Actually most genes have slightly lower expression in LON mice.

### Effects of under-nutrition on diet-induced obesity in adult mice

**Figure 3:**
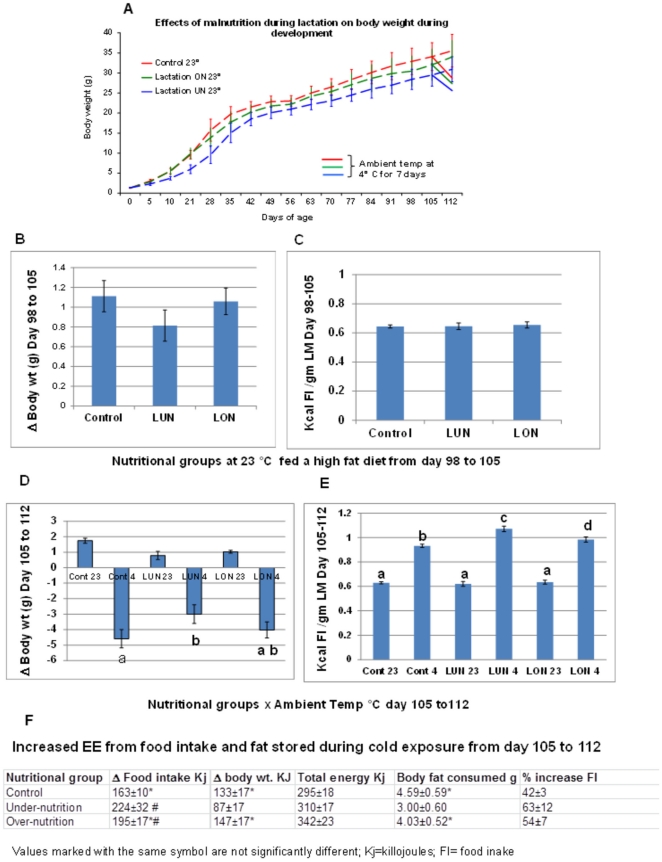
Adiposity and energy balance profiles during over- and under-nutrition. **A**. Body weight profile of C57BL/6 J mice under control, over and under-nutrition conditions. **N** = 19 for control, 23 for over-nutrition and 14 for the under-nutrition groups at 23°C. At 98 days, body weights between groups were significantly different at p<0.01. **B**. Changes in body weight in the 3 nutritional groups during the 11^th^ week on a high fat diet at 23°C; **C**. Food intake in the 3 nutritional groups at on the 11^th^ week of the high fat diet at 23°C; **D**. Changes in body weight in the 3 nutritional groups when maintained at 4°C and 23°C during the 12^th^ week. **E**. Food intake in the 3 nutritional groups during the 12^th^ week when maintained at 4°C and 23°C; **F**. Tabulation of energy balance in the 3 nutritional groups during the 12^th^ week of high diet when mice were maintained at 4°C. **The number of animals (N)** for Control, LUN and LON groups were 10, 7 and 12 respectively. For Panel B through F data bars or numbers (Panel F) with the same or no letter/symbol were not significantly different from each other at P<0.05. Data is presented as the mean ± standard error.

If the suppression of wBAT at 21 days of age by under-nutrition reduces overall thermogenesis, this may become evident in mice during induction of diet-induced obesity (DIO). We evaluated DIO in under-nourished mice under standard conditions at an ambient temperature of 23°C and subsequently when the mice were cold challenged by exposure to the cold (4°C) for 1 week (Fig. 3A). DIO was determined on 16 week old mice fed a high fat diet from 8 to 16 weeks of age and maintained at 23°C for the duration of the high fat feeding (Figure 3) or, as indicated in Figure 3A, a subgroup of mice were exposed to the cold (4°C) from week 15 to 16 (day 105 to112) while also remaining on the high fat diet. Body weight, fat mass, lean mass and food intake were measured during the course of the dietary protocol. The changes in body weight and adiposity at 23°C were very similar to those observed in previous experiments, showing that the major difference among the control, LON and LUN groups were in the reductions in diet-induced obesity in the LUN group [11]. After 105 days in the protocol, the difference in body weight and fat mass between the control and LUN group where highly significant; however, differences in body weight changes from 98 to 105 days did not reach significance Fig. 3B and food intake per g of lean mass was not different (Fig. 3C). When the ambient temperature was reduced to 4°C for 7 days a rapid reduction in body weight due to fat mass loss occurred in the 3 nutritional groups of mice (Fig. 3A and Fig. 3D). The loss in body weight was significantly less in the LUN group. Estimates of feeding efficiency indicated that food intake per gm lean mass was significantly greater in the 3 nutritional groups maintained in the cold and it was significantly higher in the LUN group than in the control and LON group (Fig. 3E). A summary of energy intake and expenditure during the week in the cold showed that while total energy expenditure was not significantly different for the 3 nutritional groups, the source of energy to maintain body temperature differed, that is, the LUN group increased energy reserves from food intake in order to consume less energy from smaller body fat stores (Fig. 3F). This may reflect a mechanism to defend a minimal fat mass.

### Expression of genes of brown adipocytes in the inguinal fat and interscapular brown fat of adult mice

**Figure 4:**
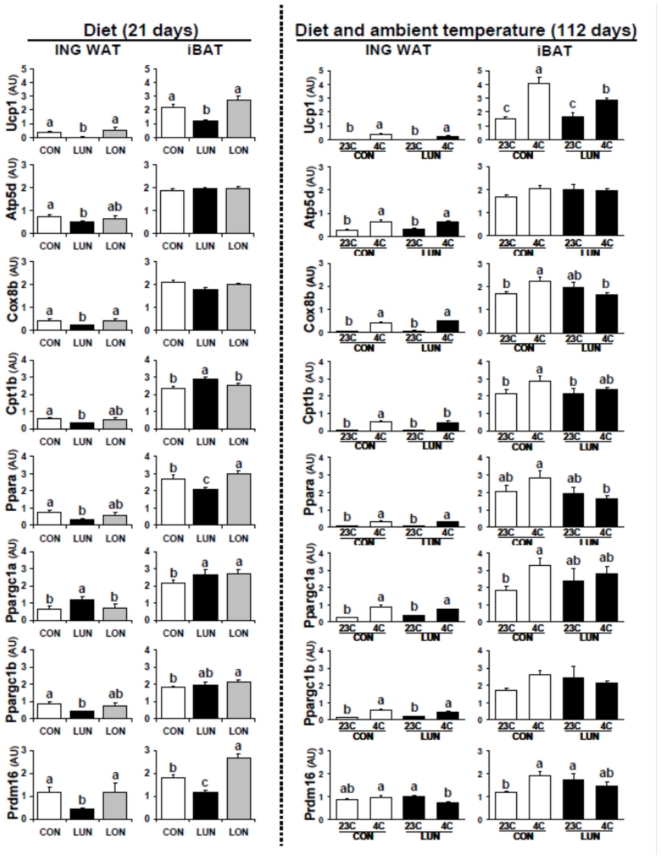
mRNA levels for genes important for brown fat function in inguinal fat and iBAT at 21 days of age after exposure to under-nutrition and over-nutrition (left-hand panel) and in 112 day old mice after 8 weeks of a high fat diet and exposure to the cold for 7 days (right-hand panel). Total RNA was isolated from tissues and analyzed by qRT-PCR using low density array with the ABI 7900 analyzer as described [32]. The data is given as the mean±standard error. The number of samples for inguinal fat was from 11–12 and for iBAT from 8–10. Significance was determined by ANOVA. Groups with the same letter were not different from each other.

The energy balance phenotypes in the 3 nutrition groups fed a high fat diet and then exposed to the cold showed that the suppression of brown adipocyte differentiation in 21 day old mice did not have long term effects on thermogenesis and DIO. LUN mice were capable of withstanding a week at 4°C indicating that their capacity for thermogenesis to defend body temperature for an extended time was not impaired. The higher level of food intake in LUN mice with reduced fat mass to lean mass, during cold exposure for 1week suggests that mice in environmental conditions that demand high energy expenditure will modulate food intake in an inverse proportion to their adiposity, rather than deplete their fat depots. The obesity and cold tolerance phenotypes suggested that brown fat thermogenesis was not impaired in adult LUN mice. We quantified the expression levels of key genes of BAT function at 21 days of age in inguinal fat and iBAT of control, LUN and LON mice. The data shows that under-nutrition suppressed expression of each of these genes in LUN mice (Fig. 4), but expression levels in control and LON mice were indistinguishable. In the iBAT modest suppression by under-nutrition was observed for *Ucp1*, PPARa and Prdm16, but several other genes were not affected (Fig. 4).With respect to the inguinal fat (Fig. 4), except for the *Lep* and *Mest* genes which showed expression that was reduced by cold exposure (data not shown), each of the BAT marker genes have similar very low levels of expression in the both Control and LUN groups at 23°C and all showed equivalent levels of induced expression following cold exposure (Fig. 4). An exception was the absence of induction by cold for *Prdm16* in inguinal fat of 112 day old mice. Thus, it appears that inguinal fat, in which under-nutrition suppressed the brown fat program at 21 days of age, was able to recover fully its ability to induce brown adipocytes in adult animals to a level similar to mice raised under control conditions. These data at the gene expression level are consistent with the adiposity phenotypes described in Fig. 3, that is, the suppression of expression of brown adipocyte genes in LUN mice at 21 days of age is not retained nor reflected in thermogenic phenotypes of adult LUN mice exposed to the cold. The lower adiposity in adult LUN mice during DIO is due to a lower capacity for adipose tissue expansion, a trait that is set between 5 and 10 days of age and which persists into adulthood [11]. Cold exposure modestly induced *Ucp1* and *Ctp1b* expression in the iBAT of mice at112 days of age (Fig. 4), but it is questionable whether this induction is biologically significant.

## Discussion

As defined by *Ucp1* mRNA levels, the magnitude of brown adipocyte induction in white fat depots of mice by cold exposure or treatment with adrenergic agonist maybe as low as 2 fold or as high as 100 fold. This variation depends upon the specific fat depot; the fold induction is higher in retroperitoneal fat and epididymal fat compared to inguinal fat. The genetic background is also a key factor, some mice such as A/J and the recombinant inbred strains AXB8 and AXB15 are high inducers, whereas others (i.e. C57BL/6J and AXB10) are low inducers [8]. Among white fat depots there is a strong correlation between the number of brown adipocytes induced and the level of *Ucp1* expression [8]. It is unknown whether the level of *Ucp1* expression/cell is higher in some brown adipocytes than others. In contrast to this high variability in the induction of wBAT, iBAT mass and its level of *Ucp1* expression is relatively stable. Under a variety of environmental conditions a 2-fold increase in cell number through cell proliferation and/or a doubling of *Ucp1* mRNA levels in iBAT occurs [16], [17]. If the mice are maintained at thermoneutrality for a period of time before cold exposure, then the overall fold induction of *Ucp1* from baseline will be a higher [18]. This comparatively low variation in iBAT expression may reflect the fact that iBAT is an indispensible thermogenic mechanism that must be fully functional immediately upon birth to ensure the survival of the newborn (note: *Ucp1−/−* mice on an inbred , as opposed to a hybrid genetic background have difficulty surviving the first few days of life at an ambient temperature of 23°C [19]). Low levels of UCP1 *i*n the mouse cannot be tolerated. On the other hand, the physiological need for the transient developmental appearance of brown adipocytes in white fat depots at weaning is not immediately obvious, nor is its rapid induction in adult mice by adrenergic stimulation. This high variability in wBAT offers an opportunity to explore the relationship between variation in brown adipocyte numbers and physiology as a consequence of nutritional variation during the early developmental period.

In an experiment primarily designed to assess the effects of malnutrition during early post-natal development on the capacity for white adipose tissue expansion, our microarray analysis of gene expression showed that under-nutrition caused a severe suppression of *Ucp1* and other genes of the brown fat differentiation program. The obvious question arose as to the effects of this suppression of brown adipocyte induction in white fat depots at weaning on the long term expression of brown adipocytes and the effects on diet-induced obesity. Surprisingly, while under-nutrition from birth to weaning strongly suppressed the transient induction of wBAT development at 21 days of age, this attenuation had no long-term effects on either diet-induced obesity or cold-induced thermogenesis in adult mice. Mice with a history of under-nutrition were able to tolerate one week in the cold at 4°C. If wBAT is a source of diet-induced thermogenesis that can reduce obesity in mice exposed to an obesogenic environment [20], then mice with suppression of wBAT should be less able to activate diet-induced thermogenesis and consequently be more obese. This clearly did not occur, since mice from the under-nourished group had reduced diet-induced obesity. Alternatively, under-nourished mice with suppressed wBAT could be less susceptible to diet-induced obesity because they would be required to use alternative thermogenic mechanisms that are calorically more costly to regulate body temperature, as we have postulated for UCP1-deficient mice [21]. Although the under-nourished mice had reduced diet-induced obesity, the cause was not from a deficiency in BAT, as shown by the similar levels of brown adipocyte gene expression in iBAT and wBAT in adult mice, irrespective of their nutrition during early development (Fig. 4). The recovery of wBAT expression in adult mice exposed to under-nutrition during early development suggests a plasticity in its expression that is perhaps derived from its inherent variability. This plasticity, that is, recovery of wBAT in adult mice, suggests that neither the severe hypoleptinemia nor hypoinsulinemia from birth to weaning have a long-term impact on the development of BAT in inguinal fat depots [11]. Furthermore, although we have no data on sympathetic nervous activity during early post-natal development, it is well known that under-nutrition suppresses the sympathetic nervous system [22]. While a reduction in sympathetic signaling could have been involved in the suppression of the wBAT phenotype at 21 days of age, it had no permanent effects on the capacity of wBAT in under-nourished mice to recover a functional BAT phenotype when subsequently fed a normal diet *ad libitum*. In short, if leptin, insulin and adrenergic signalling have effects on wBAT development and differentiation during early development, they are transient and do not affect the permanent differentiated state of the brown adipocyte in white fat depots.

A key finding of this study is that mice with suppression of wBAT at 21 days of age had recovered their normal levels of brown fat specific gene expression in the inguinal fat of the diet-induced mice at 112 days of age. The observed energy balance phenotypes discussed above are consistent with the recovery of wBAT, that is, the mice tolerated the cold, they were not obese and although the under-nourished mice were leaner than the controls and over-nourished, this phenotype is not due to the effects of wBAT. This leanness is a consequence of the fact that under-nutrition attenuates the capacity for white adipose tissue expansion , which is already well established in 10 day-old mice long before wBAT induction occurs [3], [11]. The recovery of brown fat expression in the inguinal fat of adult mice with DIO underscores the plasticity of wBAT, evident by an ability to restore normal patterns of expression following severe suppression of a phenotype by under-nutrition. This recovery of structure and function is another manifestation of plasticity of wBAT, previously observed as fat pad specific variation in wBAT [8], high inducibility by cold or β3-adrenergic agonists in adult fat depots[4], [5], [8], [23] and in the genetic variability in induction found among inbred strains of mice [8]. In contrast iBAT shows very little genetic variability [3], [24], [25], [26]. The molecular basis of the inherent instability/plasticity of wBAT, as shown by the transient nature of its expression during development and its capacity to recover completely from severe suppression by under-nutrition during early post-natal development, is of great interest to studies aimed at manipulation of wBAT thermogenic activity.

The regulatory mechanisms for induction of brown adipose tissue during development and in response to diet, hormones and ambient temperature are becoming increasingly complex.

Transgenic in vivo experiments and cell culture-based genetic manipulation have implicated at least 20 transcription and signaling factors as necessary for induction of the brown adipocyte differentiation program (for reviews see [15], [27]). The basic underlying experimental rationale for these studies posits that if over- or under expression of a candidate factor causes an induction or suppression of *Ucp1* and/or a multi-locular morphology in a cell culture or animal tissue, then evidence for a function role for this factor in BAT differentiation is in hand. Although this approach has provided plausible candidates; nevertheless; many of these transgenic models are often difficult to evaluate because the effective concentrations of transcription factors up-regulated by the powerful aP2 promoter or completely ablated by gene targeting lie outside the range of normal physiology. In some cases the quality of the phenotyping of the targeted mice does not provide unequivocal support for an increase in brown adipocyte differentiation in a tissue [15]. Equally important, at this time no plausible regulatory model has emerged that can integrate the assortment of factors revealed by the transgenic and knockout models.

Using the same rationale as that used by transgenic approaches, that is, the brown differentiation pathway is controlled by the levels of mRNA for its associated regulatory factors, we employed a QTL analysis and regression analysis of variation in mRNA expression for *Ucp1* and putative regulatory factors in the retroperitoneal fat depot of adult backcross progeny and recombinant inbred lines (RI) derived from the C57BL/6J and A/J (for review see [15]). Under conditions of cold exposure the RI lines vary as much as 100-fold in *Ucp1* levels [8]. In these genetic lines over 13 transcription factors were evaluated at the mRNA level and another 12 regulatory proteins were evaluated by immunoblots for changes in protein and/or phosphoprotein expression. RNA levels for only two transcription factors, PPARα and PGC1α, and one signaling molecule, type 2 deiodinase, were highly correlated with *Ucp1* levels (variation between the parental strains was not significantly large for most other transcription factors to be analyzed by backcross or intercross analysis) [24], [28]. Although the variation in *Pparα* and *Pgc1α* mRNA between parental B6 and A/J mice was a modest 4 to 6 fold, the variation between A/J and B6 for *Ucp1* mRNA was a robust 80 fold. However, among recombinant inbred strains, or recombinant progeny in a backcross population, the range of expression for *Pparα* and *Pgc1α* mRNA is an order of magnitude greater than that found between the progenitor strains, that is, it is similar to that found for *Ucp1* mRNA [8], [24], [28]. This genetic behavior reflects transgressive variation in which interactions among variant alleles for A/J and B6 mice generates phenotypes in recombinant animals result in phenotypes that lie outside the range observed in the parents. These genetic data indicate that synergistic interactions among unidentified QTLs determine the quantitative induction of *Ucp1*, *Pparα*, *Pgc1α* and *Dio2* mRNA and consequently the induction of brown adipocytes. The chromosomal location of the putative transcription and signaling factors identified by transgenic mice are known, however, only *Ucp1* and *Pparα* map to the locations of the QTLs that control the levels of mRNA involved in UCP1/wBAT induction [15].

The results of this earlier genetic study strongly corroborate the results in the present study on the developmental and nutritional factors that cause variation in *Ucp1* expression in wBAT. The key finding in this study is that although up to 21 genes have been associated the *Ucp1* induction in brown adipocytes, only those genes previously identified as variant in the genetic backcross analysis were linked to wBAT induction as a function of development and diet, that is, *Pparα*, *Pgc1α* and *Pgc1β*. Whereas, variation of *Dio2* mRNA was strongly associated with *Ucp1* induction in the genetic studies [28], inexplicably, in this diet/developmental study there was no association. *Prdm16* expression has not been analyzed in the backcross or RI mice; however, given that the involvement of *Prdm16* in regulation of brown adipogenesis is restricted to iBAT and inguinal wBAT [29], [30], allelic variation of *Prdm16* may not be found in retroperitoneal wBAT, consistent with the fact that *Prdm16* does not map to the QTLs. Accordingly, in 2 unbiased in vivo studies of *Ucp1* induction in wBAT, one genetic and the other nutritional, many of the transcription factors and signaling factors implicated in *Ucp1* and BAT induction by over- and under- expression in transgenic animals failed to show any significant associations. Possible explanations need to be considered. First, our *in vivo* developmental studies measuring *Ucp1* induction at 21 days of age occurred at the peak of differentiation during post-natal development. On the other hand, many of the transgenic gene KO manipulations may be acting at a much earlier stage of wBAT induction by mechanisms preceding those influenced by the nutritional environment. In other words, we need to distinguish between early developmental differentiation mechanisms and modulations in differentiated tissues. Since several of the transgenic models of brown fat induction are based upon over-expression from the aP2 promoter, assuming that the transgene is faithful to its normal in vivo expression, transgene expression will not occur until white adipocyte differentiation occurs, which is at approximately 2 days of age for inguinal fat and 6–7 days of age for visceral fat. Additionally, based upon the recent work by Seale et al. [30] demonstrating that over expression of *Prdm16* in inguinal fat led to the differentiation of the brown adipocyte phenotype, whereas over-expression of *Prdm16* in epididymal fat did not lead to wBAT, one might anticipate that iBAT and brown adipocytes in inguinal fat might share regulatory mechanisms distinct from brown adipocytes in visceral fat depots. This is consistent with the lack of genetic differences in inguinal fat between A/J and B6 mice [8]; however, the effects of developmental and nutritional variation in this study suggests that at the post-differentiation stages inguinal and visceral wBAT respond similarly to the nutritional environment. Certainly wBAT in inguinal fat shows a robust induction by cold exposure similar to wBAT in visceral fat depots. An important point to emphasize regarding QTL studies of *Ucp1* induction in RP fat by cold exposure in adult mice is that induction of *Pgc1a* and even *Ucp1* occurs within minutes of cold exposure [24]. Since *Ucp1* mRNA levels are scarcely detectable in white fat depots of adult mice at 23°C, apparently mature white adipocytes in the retroperitoneal depot may be quiescent brown adipocytes that respond to adrenergic stimulation within minutes of receiving a stimulus through epigenetic mechanisms first established at 21 days of age [31]. In conclusion, the data in this study, plus earlier QTL studies, clearly show that *Pparα*, *Pgc1α* and *Pgc1β* are the most critical transcription factors for regulation of *Ucp1*, mitochondrial biogenesis and the brown fat differentiation program in wBAT in response to variation in the nutritional and physical (i.e. temperature) environment.

We have previously shown that B6 mice double their intake of obesogenic high fat food when transferred from an ambient temperature of 28°C to 4°C, while simultaneously avoiding the normal increase in adiposity caused by the diet [34]. These profound effects of ambient temperature on preventing DIO are recapitulated in this study where a 50% reduction in fat mass was observed in mice with DIO when they were exposed to an ambient temperature of 5°C for one week. Although food intake was increased in mice exposed to the cold, the absolute amount consumed was inversely proportional to the level of adiposity at the time they were transferred to the cold. This means that more of the energy necessary to fuel thermogenesis is obtained by food intake when body fat stores are low and conversely more energy comes from body fat when fat stores are high. In any case, irrespective of the source of the energy to fuel thermogenesis, the net outcome will be the reduction of obesity, and individuals with higher obesity will lose more fat. Since it is now accepted that adult humans have measurable deposits of brown adipose tissue, there is now wide spread interest in stimulating thermogenesis to reduce obesity, mainly by drug discovery. The studies in this paper indicate that cold exposure is a simple, natural environmental strategy to prevent or reduce obesity in individuals, since humans must also utilize fat stores to fuel thermogenesis. Furthermore, physically handicapped obese individuals could be fitted with cooling water jackets to significantly increase energy expenditure in a highly controlled manner.

## Materials and Methods

### Ethics statement

The protocol #540 (Molecular Genetics of Thermogenesis) of 12/09/09 was approved by the Pennington Biomedical Research Center's Institutional Animal Care and Use Committee.

### Animals

C57BL/6J breeders were obtained from the Jackson Laboratory (Bar Harbor, Maine, USA) and maintained at Pennington Biomedical Research Center as described [32]. Newborn mice were raised from birth to weaning with one of three sets of nutritional conditions as described previously [11] 1.) the control condition had 8 pups per litter and the mother was fed the breeder diet 5015 (22 kcal % fat) *ad libitum*. 2.) Lactation under-nutrition condition (LUN) had 8 pups per litter, but the mother was only fed 50% of the food (LabDiet 5015) consumed by the control mice the previous 24 hr. 3.) Lactation over-nutrition condition (LON) had litter size reduced to 4 pups per litter and the mother was fed a high fat 58 Kcal % fat diet (Research Diet 12331, New Brunswick, New Jersey, United States) *ad libitum*. After weaning the offspring from the 3 nutritional conditions were treated the same; from weaning until 8 wk of age mice were fed a low fat chow diet (LabDiets 5053 11 Kcal % fat) *ad libitum*. At 8 wk of age mice were fed *ad libitum* a high saturated fat diet D12331 (Research Diets,) for 8 weeks. From weaning until 7 wk of age male mice were group housed (3–5 mice per pen) until 7 wk of age, at which time they were singly housed for the remainder of the experiment. All protocols were approved by the Pennington Biomedical Research Center's Institutional Animal Care and Use Committee.

### Phenotyping

Adiposity was determined from body weights and measurements of body composition by nuclear magnetic resonance (NMR, Bruker, The Woodlands, Texas, USA). RNA isolation and qRT-PCR was determined as described [33], [34]. Standard curves were generated using pooled RNA from individual samples within each experiment. Probe and primer sequences used to perform the analyses are available upon request.

### Microarray analysis

Gene expression profiles were generated using Applied Biosystems Mouse Genome Survey Microarray as previously described [11], [32]. Each microarray contained approximately 34,000 features that included a set of about 1,000 controls. Each microarray uses 32,996 probes to interrogate 32,381 curated genes representing 44,498 transcripts. Signal intensities across microarrays were normalized using the quantile-quantile method (www.bioconductor.org). The quantitative accuracy of microarray gene expression data was validated by direct comparison to the expression data obtain by analyzing the same RNA samples by qRT-PCR with TaqMan probes (see Supplemental Fig. 2 in [11]). K-means cluster analysis was conducted with Spotfire Decision Site Software (Spotfire Inc., Somerville, Massachusetts).

Microarray experiments, described according to MIAME guidelines, have been deposited in the GEO repository with the accession number GSE 19809.

### Statistical Analysis

The data are expressed as the means ± SEM. Unpaired t-test was used to compare differences between groups. Analysis of variance with Bonferroni post hoc test was used when more than two groups were compared (Statview, version 5.0.1; SAS Institute Inc., Cary, NC).
